# Development of An Individualized Risk Prediction Model for COVID-19 Using Electronic Health Record Data

**DOI:** 10.3389/fdata.2021.675882

**Published:** 2021-06-04

**Authors:** Tarun Karthik Kumar Mamidi, Thi K. Tran-Nguyen, Ryan L. Melvin, Elizabeth A. Worthey

**Affiliations:** ^1^ Center for Computational Genomics and Data Science, Departments of Pediatrics and Pathology, University of Alabama at Birmingham School of Medicine, Birmingham, AL, United States; ^2^ Hugh Kaul Precision Medicine Institute, University of Alabama at Birmingham, Birmingham, AL, United States; ^3^ Department of Anesthesiology and Perioperative Medicine, University of Alabama at Birmingham, Birmingham, AL, United States

**Keywords:** COVID-19, electronic health record, risk prediction, ICD-10, credit scorecard model

## Abstract

Developing an accurate and interpretable model to predict an individual’s risk for Coronavirus Disease 2019 (COVID-19) is a critical step to efficiently triage testing and other scarce preventative resources. To aid in this effort, we have developed an interpretable risk calculator that utilized de-identified electronic health records (EHR) from the University of Alabama at Birmingham Informatics for Integrating Biology and the Bedside (UAB-i2b2) COVID-19 repository under the U-BRITE framework. The generated risk scores are analogous to commonly used credit scores where higher scores indicate higher risks for COVID-19 infection. By design, these risk scores can easily be calculated in spreadsheets or even with pen and paper. To predict risk, we implemented a Credit Scorecard modeling approach on longitudinal EHR data from 7,262 patients enrolled in the UAB Health System who were evaluated and/or tested for COVID-19 between January and June 2020. In this cohort, 912 patients were positive for COVID-19. Our workflow considered the timing of symptoms and medical conditions and tested the effects by applying different variable selection techniques such as LASSO and Elastic-Net. Within the two weeks before a COVID-19 diagnosis, the most predictive features were respiratory symptoms such as cough, abnormalities of breathing, pain in the throat and chest as well as other chronic conditions including nicotine dependence and major depressive disorder. When extending the timeframe to include all medical conditions across all time, our models also uncovered several chronic conditions impacting the respiratory, cardiovascular, central nervous and urinary organ systems. The whole pipeline of data processing, risk modeling and web-based risk calculator can be applied to any EHR data following the OMOP common data format. The results can be employed to generate questionnaires to estimate COVID-19 risk for screening in building entries or to optimize hospital resources.

## Introduction

Despite recent progress in the Coronavirus Disease 2019 (COVID-19) vaccines approval and distribution, the pandemic continues to pose a tremendous burden to our healthcare system. Global resources to manage this current crisis continued to be in short supply. It remains critical to quickly and efficiently identify, screen and monitor individuals with the highest risks for COVID-19 so that distribution of therapeutics can be based on individual risks. Many factors including pre-existing chronic conditions (Liu et al., 2020), age, sex, ethnicity and racial background, access to health care, and other social-economic components (Rashedi et al., 2020) have been shown to affect an individual’s risk for this disease.

Accordingly, several predictive models that seek to optimize hospital resource management and clinical decisions have been developed (Jehi et al., 2020a; Jehi et al., 2020b; Gong et al., 2020; Liang et al., 2020; Wynants et al., 2020; Zhao et al., 2020). To a large degree, these informatic tools leverage the vast and rich health information available from Electronic Health Record (EHR) data (Jehi et al., 2020b; Oetjens et al., 2020; Osborne et al., 2020; Vaid et al., 2020; Wang et al., 2021a; Wang et al., 2021b; Estiri et al., 2021; Halalau et al., 2021; Schwab et al., 2021). EHR systems contain longitudinal data about patients’ demographics, health history, current and past medications, hospital admissions, procedures, current and past symptoms and conditions. Although the primary purpose of EHRs is clinical, over the last decade researchers have used them to conduct clinical and epidemiological research. This has been notable especially during the COVID-19 pandemic where such research that generated invaluable data about COVID-19 risks, comorbidities, transmission and outcomes was quickly adapted for clinical decision making (Daglia et al., 2021). To ensure interoperability across multiple hospital systems, EHR data incorporate standard reference terminology and standard classification systems such as the International Classification of Diseases (ICD) that organize and classify diseases and procedures for facile information retrieval (Bowman, 2005). Incorporated into the Medical Outcomes Partnership (OMOP) common data model (Blacketer, 2021), these ICD9/ICD10 codes facilitate systemic analyses of disparate EHR datasets across different healthcare organizations.

Many of these insights were generated using machine learning methods, based on multi-dimensional data (Mitchell, 1997). Studies have employed a variety of classification and/or regression methods including Naive Bayes, Support Vector Machine, Decision Tree, Random Forest, AdaBoost, K-nearest-neighbor, Gradient-boosted DT, Logistic Regression, Artificial Neural Network, and Extremely Randomized Trees (Alballa and Al-Turaiki, 2021). Among these, the most popular methods applied to COVID-19 have been linear regression, XGBoost, and Support Vector Machine (Alballa and Al-Turaiki, 2021).

To develop a COVD-19 risk model, we chose a Logistic Regression based Credit Scorecard modeling approach to estimate the probability of COVID-19 diagnosis given an individual’s ICD9/ICD10 encoded symptoms and conditions. Credit Scorecard is a powerful predictive modeling technique widely adopted by the financial industry to manage risks and control losses when lending to individuals or businesses by predicting the probability of default (Bailey, 2006). The Credit Scorecard model is most frequently used by scorecard developers not only due to its high prediction accuracy, but also due to its interpretability, transparency and ease of implementation. This method has been implemented previously for EHR data based COVID-19 risk prediction (Jehi et al., 2020a; Jehi et al., 2020b).

Application of feature selection methods that attempt to retain the subset of features that are most applicable for classification has been applied to increase interpretability, enhance speed, reduce data dimensionality and prevent overfitting (Alballa and Al-Turaiki, 2021). While there are many feature selection methods, sparse feature selection methods such as LASSO (Least Absolute Shrinkage and Selection Operator) (Tibshirani, 1996) and Elastic-Net (Zou and Hastie, 2005) provide advantages. LASSO places an upper bound constraint on the sum of the absolute values of the model parameters by penalizing the regression coefficients based on their size and forcing certain coefficients to zero and eventually excluding them to retain the most useful features (Tibshirani, 1996). Expanded from LASSO, Elastic-Net adds a quadratic penalty term to the calculation of coefficients to prevent the “saturation” problem encountered when a limited number of variables are selected (Zou and Hastie, 2005). Several COVID-19 risk prediction models employed LASSO (Gong et al., 2020; Liang et al., 2020; Feng et al., 2021) and Elastic-Net (Heldt et al., 2021; Hu et al., 2021; Huang et al., 2021).

The major goals for this analysis were to determine whether we could: 1) leverage the existing hierarchical structure of the ICD9/ICD-10 classification system, in an unbiased approach, to capture patients’ symptoms and conditions and estimate their possibilities of having a COVID-19 diagnosis, 2) examine the temporal aspect of EHR (i.e., within a timeframe, for example, symptoms within 2-weeks of infection/diagnosis). to evaluate what current symptoms and/or pre-existing conditions affect COVID-19 risks, 3) apply a Credit Scorecard modeling approach to develop and validate a predictive model for COVID-19 risk from retrospective EHR data, and 4) develop a pipeline requiring minimal manual curation capable of generating COVID-19 risk models from any EHR data using the OMOP common data model (Blacketer, 2021). To demonstrate the latter goal a web application was created to take answers from individuals and produces a COVID-19 risk score. We have made the code freely available for anyone wishing to reproduce and deploy such a model at gitlab.rc.uab.edu/center-for-computational-genomics-and-data-science/public/covid-19_risk_predictor.

## Materials and Methods

### Dataset

The UAB Informatics Institute Integrating Biology and the Bedside (i2b2) COVID-19 Limited Datasets (LDS) contain de-identified EHR data that are also part of the NIH COVID-19 Data Warehouse (NCATS, 2020). Data was made available through the UAB Biomedical Research Information Technology Enhancement (U-BRITE) framework. Access to the level-2 i2b2 data was granted upon self-service pursuant to an IRB exemption. Our dataset contains longitudinal data of patients in the UAB Health System who had COVID-19 testing and/or diagnosis from January to June 2020. Aggregated from six different databases, our dataset was transformed to adhere to the OMOP Common Data Model Version 5.3.1 (Blacketer, 2021) to enable systemic analyses of EHR data from disparate sources.

The UAB i2b2 COVID-19 LDS is comprised of 14 tables corresponding to different domains: PERSON, OBSERVATION_PERIOD, SPECIMEN, DEATH, VISIT_OCCURRENCE, PROCEDURE_OCCURENCE, DRUG_EXPOSURE, DEVICE_EXPOSURE, CONDITION_OCCURENCE, MEASUREMENT, OBSERVATION, LOCATION, CARE_SITE and PROVIDER. For the purpose of this study, we limit assessment to previous reported conditions (from CONDITION_OCCURENCE) and lifestyle/habits (from OBSERVATION).

### Data Processing

Data wrangling was performed using Python 3.8.5 with the Pandas package 1.2.1 and Numpy package 1.19.5. Code for recreating our process is freely available (see code availability statement below). The following subsections detail the information retrieved from the database tables mentioned above.

### Person Table

Demographic information (i.e., age, gender, race, and ethnicity) for each de-identified individual was extracted from the PERSON table. Ages were extracted using the “year of birth” values.

### Measurement Table

Information about COVID-19 testing was stored in the Measurement table. We extracted the date, test type and test result for each person.

COVID-19 positivity was determined by the presence of either one of the three criteria: positive COVID-19 antibody test, positive COVID-19 Polymerase Chain Reaction (PCR) test, or the presence of ICD-10 U07.1 code in the EHR record. COVID-19 negativity was assigned if the person were tested for COVID-19 but has never had a positive test nor an ICD-10 U07.1 code.

### Condition_Occurence Table

We extracted medical conditions (such as signs and symptoms, injury, abnormal findings and diagnosis) for each patient from this table by leveraging the inherent hierarchical structure of the ICD-10 classification system.

### Observation Table

Lifestyle and habits (i.e., BMI, smoking, alcohol and substance use) were extracted from this table. This table also includes the current status (i.e., current, former, never or unknown) of habits for each patient.

### Feature Filtering and Extraction

Demographics, lifestyle/habits and conditions (encoded by ICD-9/ICD-10) are obtained as features in our model. For the purpose of using the updated version of ICD codes as features, we converted all ICD-9 codes to ICD-10 codes using a publicly available converter script (Hanratty, 2019). We used these converted codes along with the original ICD-10 codes to map and extract conditions reported in the EHR for each patient.

Before feature extraction, we filtered out all COVID-19 related ICD-10 codes such as U07.1 (COVID-19, virus identified), Z86.16 (personal history of COVID-19), J12.82 (pneumonia due to coronavirus disease 2019), B94.8 (sequelae of COVID-19), B34.2 (Coronavirus infection, unspecified), and B97.2 (Coronavirus as the cause of diseases classified elsewhere). Discarding COVID-19-related codes is imperative to prevent data leakage in our predictive model. Data leakage refers to the inclusion of information about the target of the prediction in the features used for making the prediction that should not be (legitimately) available at the time a prediction is made (Huang et al., 2000; Nisbet et al., 2009; Kaufman et al., 2012; Filho et al., 2021).

### Temporal Filter for Medical Condition data

For the positive cohort, we used the date of patients’ first COVID-19 testing or their first assignment of the COVID-19-related ICD-10 codes (U07.1, U07.2, Z86.16, J12.82, B94.8, B34.2, or B97.29) as the timestamp to apply a temporal filter for feature selections. For the negative cohort, we also used the date of their first COVID-19 testing as the timestamp. We define temporal filter as a restricted timeframe to study the effect of conditions for infection (i.e., to assess risk using medical conditions occurred within 2 weeks before an infection). This temporal filter is crucial to once again avoid data leakage by excluding features that may emerge as a result of a COVID-19 infection or diagnosis.

To investigate how the timing of medical events and conditions may affect the risk for COVID-19, we extracted the condition data over two distinct time intervals. The first timeframe only considers the conditions within the 2-week window prior to the date of diagnosis whereas the second timeframe retains all condition data before a given patient’s first COVID-19 test or diagnosis.

### Credit Scorecard Model

#### Variable (Feature) Selections

After extracting patients’ demographic information, lifestyle, habits and ICD-10 condition codes, we converted them to features using one-hot encoding. Features with more than 95% missing data or 95% identical values across all observations were removed. The remaining variables underwent weight-of-evidence (WoE) transformation, which standardizes the scale of features and establishes a monotonic relationship with the outcome variable (Zdravevski et al., 2011). WoE transformation also handles missing and extreme outliers while supporting interpretability through enforcing strict linear relationships (Zdravevski et al., 2011). WoE transformations require all continuous or discrete variables to be binned. This binning process is carried out programmatically based on conditional inference trees (Hothorn et al., 2006). Missing values for each feature are placed in their own bin and eventually assigned their own WoE values. Each level (x) of the binned values for each feature is then assigned a WoE value via $WoE\left(x\right)=\ln\left(\frac{P\left(x\text{|}y=1\right)}{P\left(x\text{|}y=0\right)}\right)$ where P(x/y) is the conditional probability of x given y, and y is the binary response variable. All values of the independent variables, including missing values, are then replaced with their corresponding WoE value (Zdravevski et al., 2011; Szepannek, 2020). These transformed variables were then used in logistic regression to assign weights for the Scorecard.

For feature selection and regression on these transformed variables, we tested two regularization approaches, LASSO (Tibshirani, 1996) and Elastic-Net (Zou and Hastie, 2005), using a cross-validation-based logistic regression method from the Python package *Scikit-Learn* (version 0.23.2). This method incorporates the use of stratified cross-validation to determine optimal parameters for LASSO and Elastic-Net. LASSO is a modification to typical generalized linear modeling techniques such as logistic regression. Under the constraint the sum of the absolute value of the model coefficients are less than a constant, the residual sum of square errors is minimized (Tibshirani, 1996). The application of this constraint results in some coefficients being 0, making LASSO a simultaneous variable selection and model fitting technique. Building on LASSO, Elastic-Net adds a quadratic penalty term to the calculation of coefficients. Practically, this additional term prevents the “saturation” (Zou and Hastie, 2005) problem sometimes experienced with LASSO where an artificially limited number of variables are selected. Both techniques employ penalty terms to shrink variable coefficients to eliminate uninformative features and avoid collinearity.

Collinearity is a major problem in extracting features from ICD codes since some codes are frequently reported together, or different providers may use inconsistent and incomplete codes. Between the two approaches, LASSO is a more stringent variable selector. For example, in the case of two highly similar features, LASSO tends to eliminate one of them while Elastic-Net will shrink the corresponding coefficients and keep both features (Hastie et al., 2001).

The regularization strength (for both LASSO and Elastic-Net) parameter and mixing parameter (for Elastic-Net) were selected using 10-fold stratified cross-validation (CV). This method creates 10 versions of the model using a fixed set of parameters, each trained on 90% of the training data with 10% held out in each “fold” for scoring that particular instance of the model. The stratified variant of CV ensures that the distribution of classes (here COVID-positive patients and COVID-negative patients) is identical across the 90%/10% split of each fold. This process enables the model developer to assess the predictive capability of the model given the specific set of parameters being tested. The scores over all folds are averaged to assign an overall score for the given set of parameters. This process is repeated for all candidate sets of parameters being tested. Cross-validation aids in preventing overfitting, i.e., failing to generalize the pattern from the data, because the model is judged based on its predictions on hold-out data, which are not used for training the model.

For scoring candidate sets of parameters, we chose negative log loss, a probability-based scoring metric, because a Scorecard model is based on probabilities rather than strict binary predictions. In particular, negative log loss penalizes predictions based on how far their probability is from the correct response (Bishop, 2016). For example, consider a patient who is in truth COVID-negative. A forecast that a COVID-positive diagnosis is 51% likely will be penalized less harshly than a forecast that COVID-positive is 99% likely. Conversely, a forecast that a positive diagnosis is 49% likely will be rewarded less than one that such a diagnosis is 1% likely.

The hyperparameters evaluated for candidate LASSO models was regularization strength, or the inverse of lambda referred to in (Tibshirani, 1996). One-hundred candidate values on a log scale between 1e^−4^ and 1e^4^ were considered. The model with the best score from the technique described above was considered to have the optimal hyperparameters. For Elastic-Net, the same set of regularization strength parameters was considered. Additionally, Elastic-Net has a mixing parameter that controls the relative strength of the LASSO-like penalty and the additional Elastic-Net penalty term. Ten evenly spaced values between 0 and 1 were considered for this hyperparameter.

To address the class imbalance between COVID-19 positive and negative group in the training data, we weighted each observation inversely proportional to the size of its class. Likewise, the use of a stratified cross-validation method reduces the risk of inflating some scoring metrics by the model preferring to simply predict the dominant class. Using the above methods, we wanted to compare and contrast four models to predict the risk for infection. Below are the four models:

1. LASSO with all conditions/features reported before the infection/diagnosis
2. Elastic-Net with all conditions/features reported before the infection/diagnosis
3. LASSO with only conditions/features reported within 2 weeks of infection/diagnosis
4. Elastic-Net with only conditions/features reported within 2 weeks of infection/diagnosis

#### Model Evaluations

Data were randomly split into 80% for the train set and 20% for the test set. The quality of the four models built from two different time-filtered datasets and two different regularization techniques were evaluated by plotting the Receiving Operating Characteristic (ROC) curve and measuring the corresponding Area Under the ROC Curve (AUC). We also considered other model quality metrics such as Accuracy (ACC)—the percent of correct responses—and F-score—the harmonic mean of precision and recall. We also used the confusion matrices to judge the quality of our candidate models. Considering that these models are built to recommend COVID-19 testing, we sought to avoid False Negative predictions while being more lenient towards False Positive errors.

#### Risk Score Scaling Using the Scorecard Method

Coefficients from the resulting logistic regression models were then combined with the WoE-transformed variables to establish scores for each feature in the Scorecard. This scorecard generation was performed using the Scorecard method implemented in the *scorecardpy* python package (version 0.1.9.2). As opposed to pure logistic regression models, scorecard models allow a strictly linear combination of scores that can be calculated even on a piece of paper, without the aid of any technology. Calculating the probabilities from a logistic regression model would require inverse transformations of log odds. We chose the scorecard model for the strict linear interpretation and corresponding ease of deployment anywhere.

This method requires users to select target odds and target points (a baseline number of points corresponding to a baseline score) along with the points required to double the odds. As these choices are arbitrary, we used the package defaults, which set the target odds to 1/19, the corresponding target points to 600, and the default points required to double the odds to 50. Supplemental Figure S1 shows an example of a Scorecard distribution calculated in this manner. Since the final Scorecard model is a linear function of the predictors (i.e., higher scores indicate higher COVID-19 risks), using scorecards has many benefits such as transparency, interpretability and facile implementation.

### Building a Web Application to Predict COVID-19 Risks

Based on the final Scorecard model results, we used the *streamlit* package (version 0.77.0) in Python to build an interface and used interactive indicator plot from *plotly* to visualize the risk score. The Python code to build this application can be found in our gitlab repository at gitlab.rc.uab.edu/center-for-computational-genomics-and-data-science/public/covid-19_risk_predictor.

## Results

Our dataset was composed of 7,262 patients from within the UAB Health System who received COVID-19 testing or diagnosis from January to June 2020. The demographic information of this study population is shown in Table 1. Among them, 912 patients were diagnosed with COVID-19 and the remaining 6,350 patients, were not. On average, patients in the positive group received 13% more COVID-19 tests (1.45 vs. 1.19 tests/person). While there is no statistically significant difference in age and gender between the two groups, African American (46 vs. 39%), Asian (3 vs. 1%) and Others (11 vs. 3%) ethnicity were overrepresented in the positive group, a finding which is concordant with other reports about the racial disparity in COVID-19 (Kullar et al., 2020). In this UAB Health System dataset, a greater number of patients in the negative group reported substance abuse (14 vs. 3%) and current smoking (25 vs. 9%). There was no difference in Body Mass Index (BMI) between the two groups. Although the COVID-19 negative group had more reported medical conditions (178 vs. 142 medical conditions/person), they had fewer unique medical conditions (4 vs. 10 unique conditions/person).

**TABLE 1:** Demographics and Clinical Characteristics of the UAB LDS N3C Cohort.

UAB LDS N3C cohort (*n* = 7,262)		
COVID-19 testing:		
COVID-19 results	Positive (*n* = 912)	Negative (*n* = 6,350)
Total COVID tests	1,328	7,596
COVID Tests/Person	1.46	1.20
All medical tests:		
All tests	1,951,404	17,395,613
All tests/person	2,139	2,739
Age	mean = 52 (10–119)	mean = 52 (<1–119)
Gender:		
Male (%)	394 (43%)	3,035 (48%)
Female (%)	516 (57%)	3,314 (52%)
Unknown (%)	2 (0%)	1 (0%)
Race:		
White (%)	337 (37%)	3,441 (54%)
Black (%)	416 (46%)	2,497 (39%)
Asian (%)	27 (3%)	70 (1%)
Hispanic (%)	28 (3%)	174 (3%)
Others (%)	104 (11%)	168 (3%)
Conditions:		
Total conditions	129,091	1,133,396
Unique conditions	9,224	24,101
#Conditions/Person	142	178
#Unique conditions/Person	10	4
Smoking:		
Current smoker	81 (9%)	1,602 (25%)
Former smoker	196 (21.5%)	1,625 (26%)
Never smoker	368 (40%)	2,589 (41%)
Unknown	13 (1%)	64 (1%)
Substance use:		
Current substance abuse	27 (3%)	895 (14%)
No substance abuse	632 (69%)	4,716 (74%)
Former substance abuse	32 (3.5%)	402 (6%)
Unknown	15 (1.6%)	74 (1%)
Alcohol use:		
Current alcohol	273 (30%)	1954 (31%)
Former alcohol	58 (6%)	652 (10%)
No alcohol	379 (41.5%)	3,459 (54.5%)
Unknown	12 (1.3%)	80 (1%)
Weight:		
Underweight (BMI < 19)	20 (2%)	271 (4%)
Normal weight (BMI = 20–25)	49 (5%)	563 (9%)
Overweight (BMI = 25–40)	320 (35%)	2,439 (38%)
Obese (BMI > 40)	120 (13%)	773 (12%)

**FIGURE 1:**
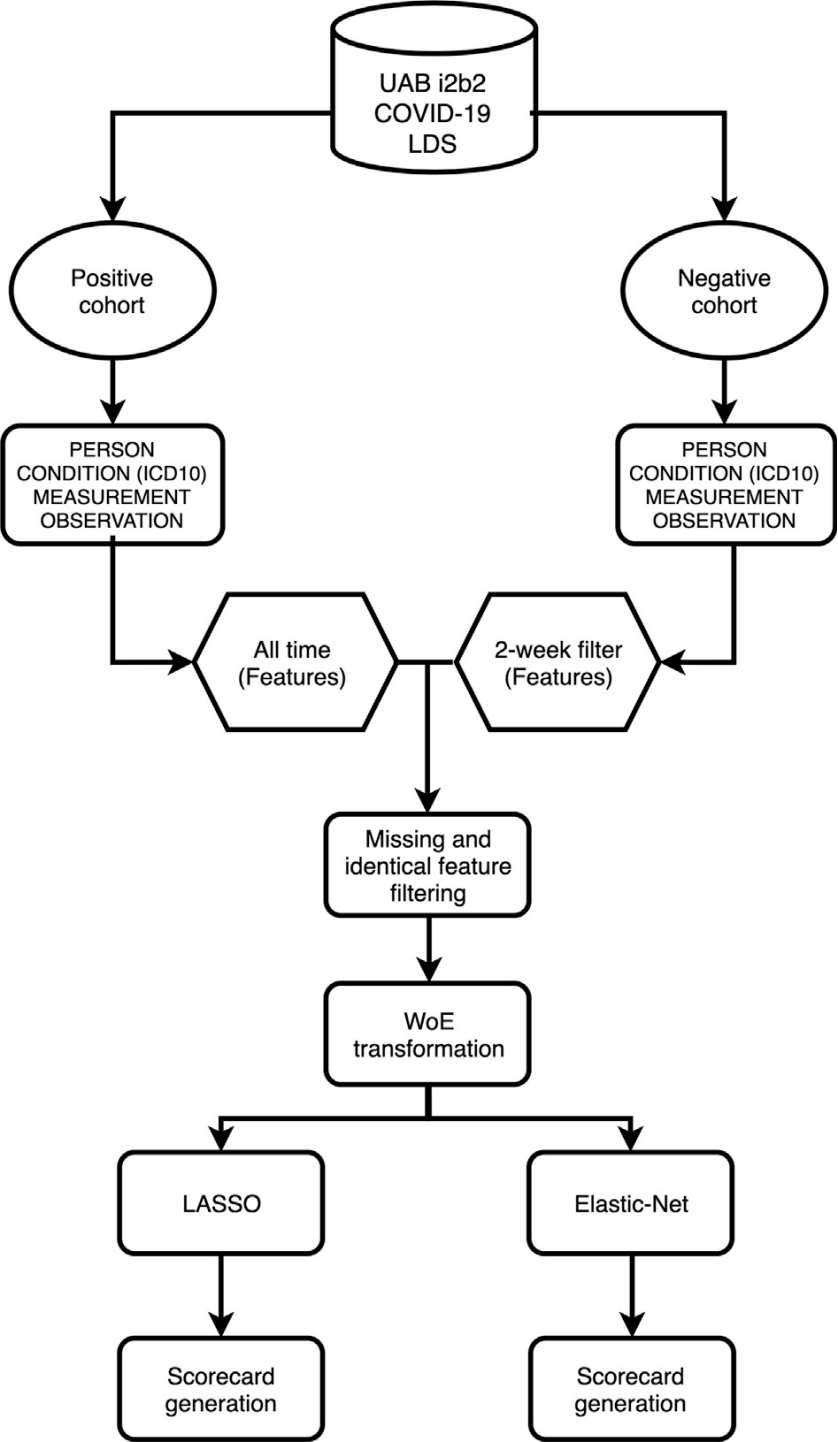
Overview of workflow.

**FIGURE 2:**
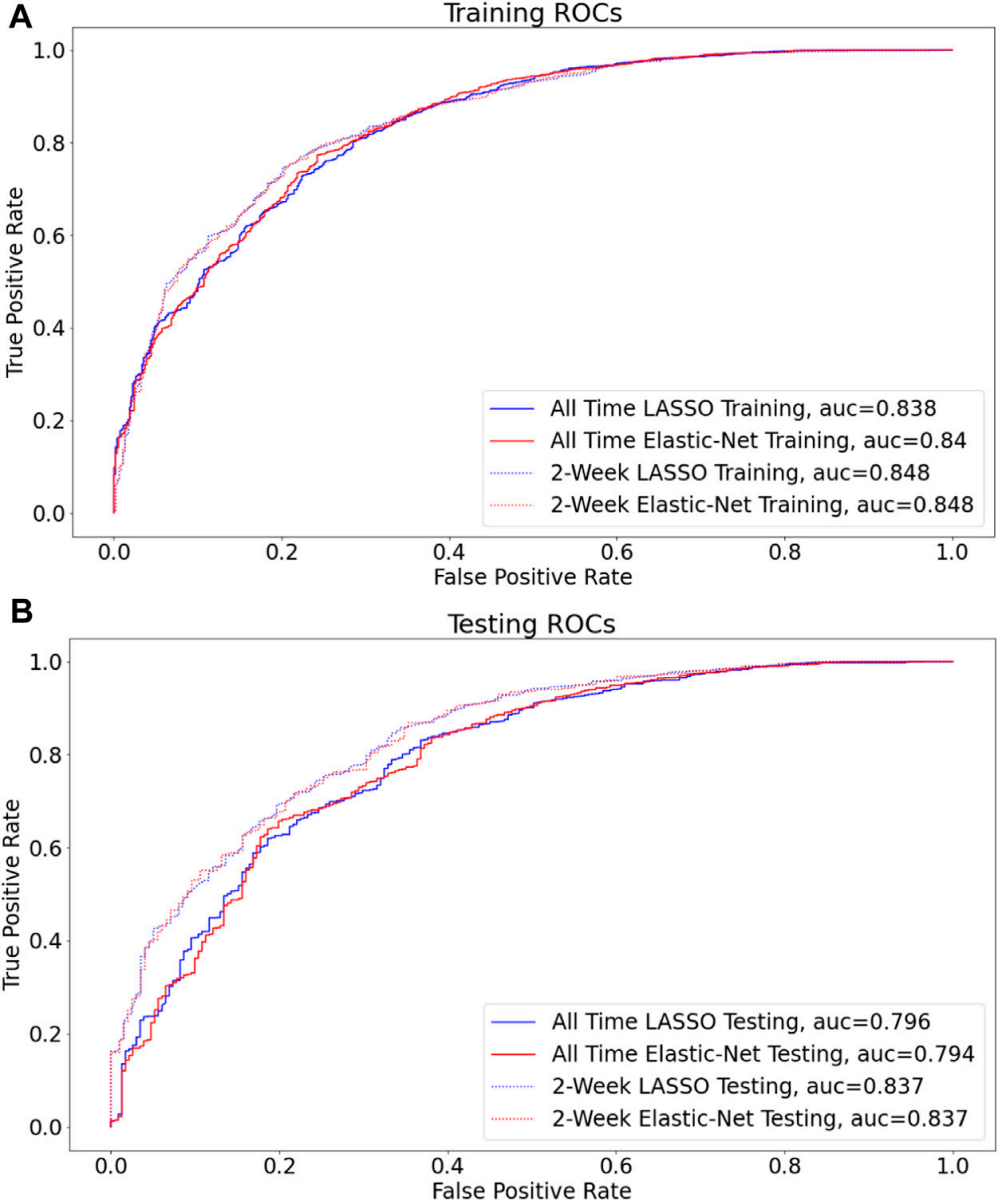
LASSO vs Elastic-Net model performance on two sets of data Receiver operating characteristic (ROC) curves are shown for the final model for each of the four assessed techniques **(A,B)**, and the corresponding areas under curves (AUC) are presented in the figure legend. By AUC on hold out data (0.815), the models built on data filtered by two-week before COVID (non)diagnosis perform the best (B).

The workflow to build the predictive model for COVID-19 diagnosis based on EHR data is summarized in Figure 1. We used condition data extracted from ICD-9/ICD-10 codes from two different timeframes to assess how the timing of medical symptoms and conditions may affect our COVID-19 risk predictions. The first timeframe considers the data reported within a 2-week window of testing/diagnosis while the second timeframe retains all condition data prior to a COVID-19 test or diagnosis. Such condition data suffer from collinearity issues in that a group of medical conditions tends to be reported together, and different providers may use inconsistent codes for the same conditions. To address these collinearity issues, we utilized two different regularized regression techniques, LASSO and Elastic-Net. Applying these two methods on the two data timeframes yielded four different models with reasonable discriminatory power, as judged by performance metrics on testing data. With LASSO, we achieved 0.75 accuracy and 0.84 [CI: 0.81–0.87] AUC for the 2-week data and 0.74 accuracy and 0.80 [CI: 076–0.83] AUC for all-time data (Figure 2; Table 2). Elastic-Net models also performed with a similar accuracy of 0.76 and AUC of 0.84 [CI: 0.81–0.87] for the 2-week data and an accuracy of 0.74 and AUC of 0.79 [CI: 0.76–0.83] for the all-time data (Figure 2; Table 2).

**TABLE 2:** Model metrics Evaluation of four models (LASSO and Elastic-Net with patient’s conditions information from two timeframes) while training and testing (i.e., holdout) data set. For each model, the accuracy, F-Score, and AUC with 95% CI using DeLong’s method (DeLong et al., 1988) are shown. The accuracy metric indicates the percent of correct predictions. F-score is the harmonic mean of precision and recall. Area under receiver operating curve (AUC) is the area under the curve resulting from plotting the true positive against the false positive rate.

Training metrics			
All-Time + LASSO		All-Time + Elastic-Net	
Accuracy	0.746	Accuracy	0.755
F-Score	0.834	F-Score	0.840
AUC	0.838	AUC	0.840
95% AUC CI	[0.82 0.86]	95% AUC CI	[0.82 0.86]

2-Week + LASSO		2-Week + Elastic-Net	
Accuracy	0.774	Accuracy	0.775
F-Score	0.847	F-Score	0.848
AUC	0.848	AUC	0.848
95% AUC CI	[0.83 0.87]	95% AUC CI	[0.83 0.87]

Testing Metrics			
All-time + LASSO		All-time + Elastic-Net	
Accuracy	0.741	Accuracy	0.744
F-Score	0.832	F-Score	0.834
AUC	0.796	AUC	0.794
95% AUC CI	[0.76 0.83]	95% AUC CI	[0.76 0.83]

2-Week + LASSO		2-Week + Elastic-Net	
Accuracy	0.753	Accuracy	0.755
F-Score	0.833	F-Score	0.835
AUC	0.837	AUC	0.837
95% AUC CI	[0.81 0.87]	95% AUC CI	[0.81 0.87]

Using LASSO, a more stringent regularization method where many variables are eliminated through shrinkage, after filtering, 30 out of the 58 features were retained (Supplemental Table S1) in the 2-week data, and 93 out of 212 features were retained in the all-time data (Supplemental Table S2). Within two weeks before a COVID-19 diagnosis, features that predict higher risks for this disease were cough (R05), abnormalities of breathing (R06), pain in throat and chest (R07), abnormal findings on diagnostic imaging of lung (R91), respiratory disorder (J98), disorders of fluid, electrolyte and acid-base balance (E87), nicotine dependence (F17), major depressive disorder (F32) and overweight and obesity (E66) (Supplemental Table S1). The LASSO model on all-time data identified similar features from the 2-week data such as cough (R05), but it also delineated other important features related to acute respiratory infections such as fever (R50), pain (R52), acute upper respiratory infections (J06), respiratory failure (J96), respiratory disorder (J98), pneumonia (J18), vasomotor and allergic rhinitis (J30), and other disorders of nose and nasal sinuses (J34). Most notably, the all-time model uncovered several chronic conditions in other organ systems besides the respiratory system including neurological disorders e.g. postviral fatigue syndrome (G93, R41), kidney diseases (I12, I13, N17), diseases of the heart and circulation including hypertension and kidney failure (I49, I51, J95) and fibrosis/cirrhosis of the liver (K74), suggesting that long-term chronic conditions in other organ systems may increase the risks for contracting an acute respiratory illness such as COVID-19.

Even though LASSO is an effective method to handle collinearity issues, it may not work well with multicollinearity where several features are correlated among each other, as observed in our condition data. Considering that LASSO may eliminate important features through the stringent shrinkage process, we also implemented the Elastic-Net regularization method as a less stringent variable selector. This approach retained more features than the LASSO with 43 features remained for the 2-week data and 179 features for the all-time data. All features selected from the LASSO method also remained in the Elastic-Net method. Several new predictive features emerged from the 2-week data including primary hypertension (I10) and gastro-esophageal reflux disease (K21). In the all-time data, many distinct yet similar conditions from the LASSO model also appeared such as acute myocardial infarction (I21), cardiomyopathy (I42), other cardiac arrhythmias (I49), cerebral infarction (I63), complications and ill-defined descriptions of heart disease (I51), peripheral vascular diseases (I73), and other cerebrovascular diseases (I67), pointing to vascular disorders. Other medical conditions also emerged including viral hepatitis (B19), bacterial infection (B96), thrombocytopenia (D69), epilepsy and recurrent seizures (G40), although the predictive powers of these variables were low.

**FIGURE 3:**
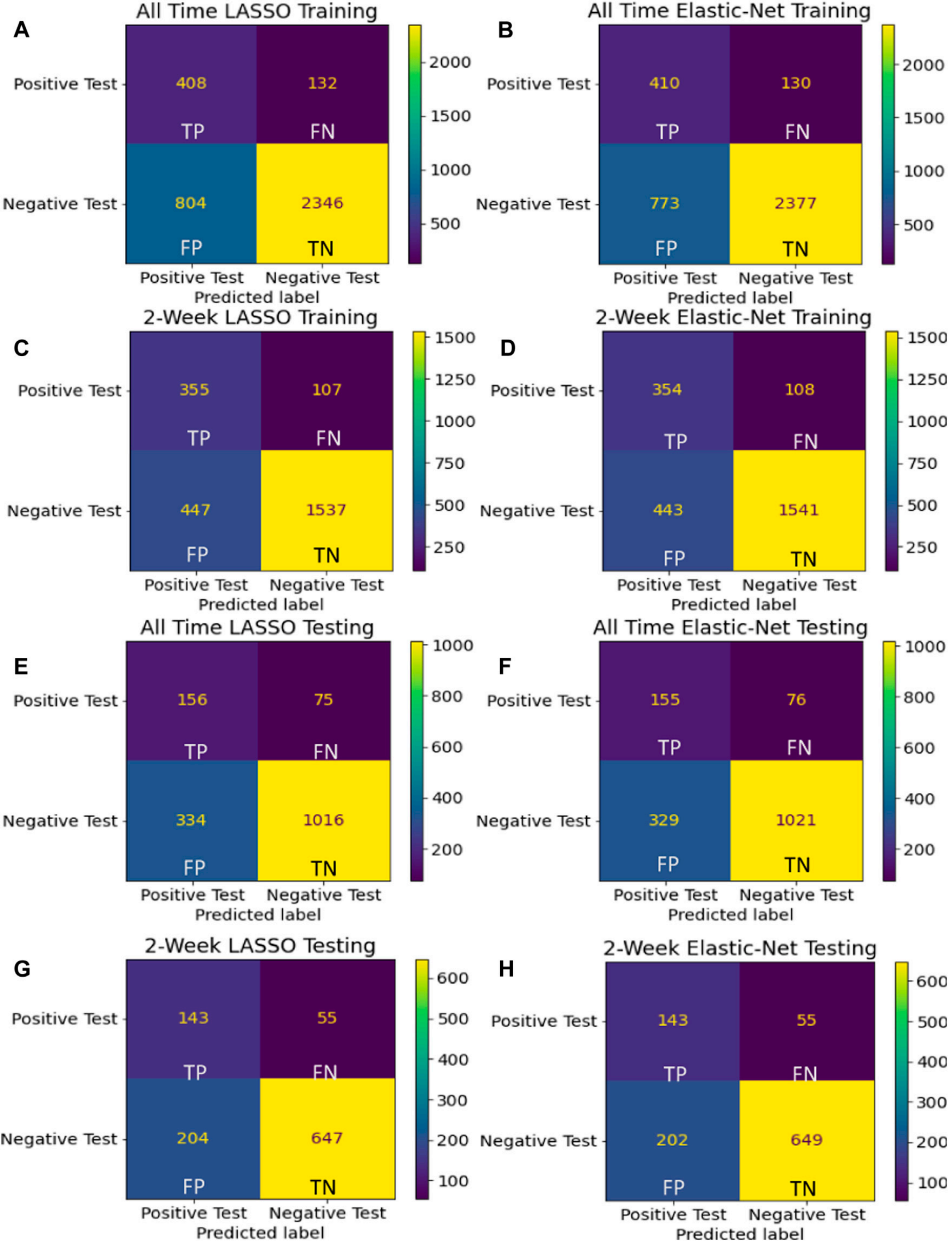
Confusion matrices Confusion matrices using training **(A–D)** and holdout **(E–H)** data are shown for the final model for each of the four assessed techniques. Considering that these models are built to recommend COVID-19 testing, we sought to avoid False Negative predictions while being more lenient towards False Positive errors.

**FIGURE 4:**
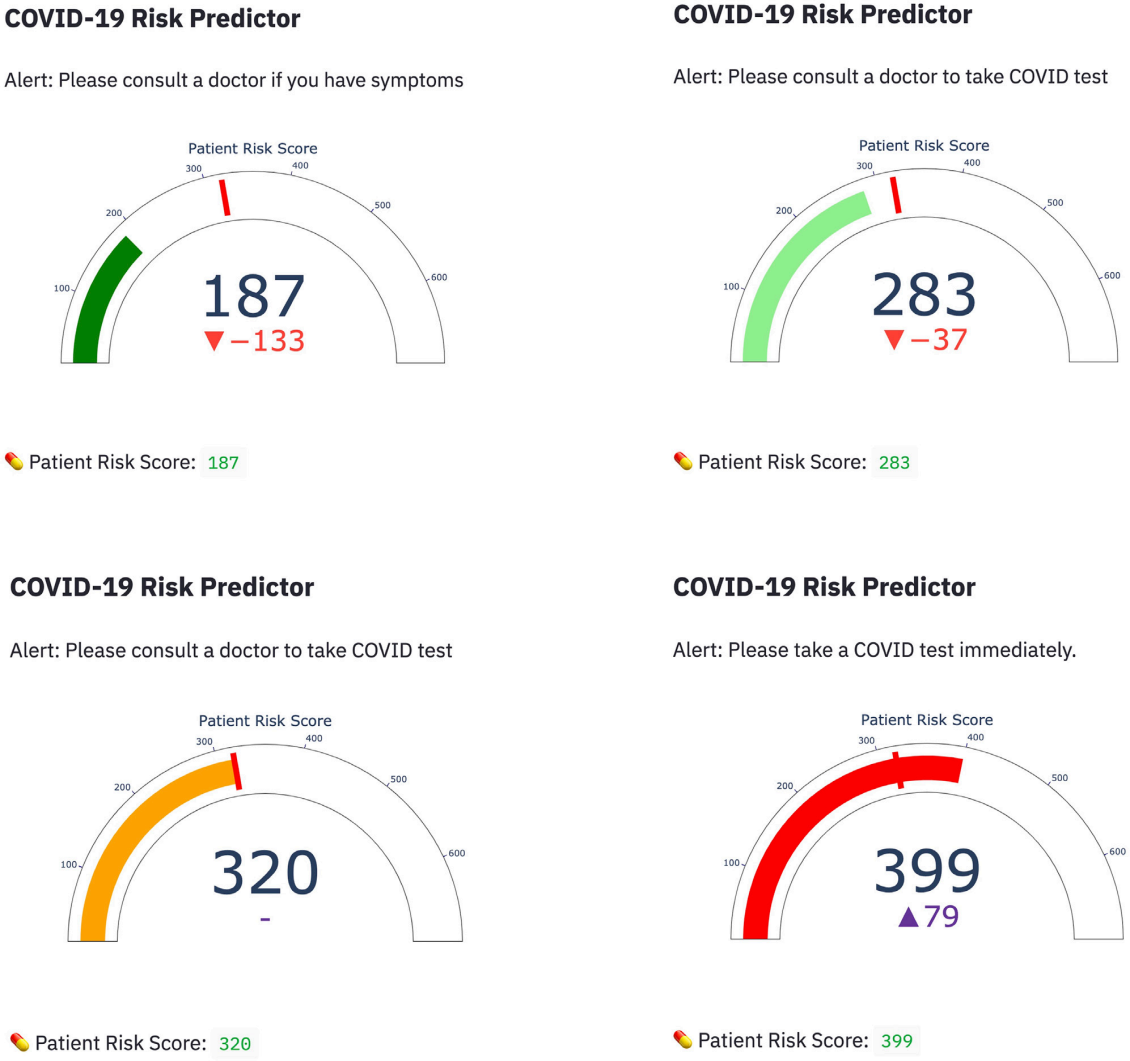
Web application demonstration Four representative snapshots with different scorings from the COVID-19 risk predictor web application are shown. Scores were calculated based on participant answers to questions related to their symptoms and conditions using the Credit Scorecard method.

Among the four candidate models we generated based on the UAB-i2b2 data, the LASSO method on the 2-week filtered data retained the fewest variables while achieving similar performance with other more complex models (Figures 2, 3; Table 2; Supplemental Tables S1–S4). For this reason, we believed this is a superior model and selected it as the model for our web application. This interactive web application (Figure 4) gathers participant questionnaire inputs and generates a risk prediction score of having COVID-19. The Scorecard distribution based on the logistic regression model can be found in Supplemental Figure S1. This tool can be used for individuals to check their risks based on their symptoms or conditions, or for organizations to build questionnaires to perform COVID-19 screening for building entries. An example questionnaire from our final model is provided in Table 3.

**TABLE 3:** Example questionnaire Example questionnaire built using our selected model using the UAB-i2b2 data—the LASSO method on the 2-week filtered data. Base score is 320 and the risk increases/decreases based on the answers in the questionnaire. Any score between 450 and 696 is considered high risk for infection. Disclaimer: This questionnaire is intended only as an example output from a model built using our pipeline. It is not itself a diagnostic tool.

Questions	Yes	No
Do you have chronic kidney disease?	36	−6
Do you have cough?	36	−44
Have you delivered a baby?	35	−2
Are you having acute upper respiratory infections?	30	−6
Do you have fever?	24	−5
Are you having depression, anxiety, problems with cognitive functions or other brain disorders?	17	−4
Are you having pneumonia?	17	−3
Are you having respiratory failure?	16	−3
Are you dependent on nicotine?	14	−4
Do you have allergic rhinitis?	14	−2
Do you have retention of urine?	14	−1
Do you have pain?	14	−1
Do you have hernia?	13	−1
Do you have liver fibrosis/cirrhosis?	13	−1
Do you have disturbances of skin sensation?	12	−2
Are you having anemia?	10	−1
Are you having bacterial infection?	9	−1
Do you have complications from heart disease?	8	−2
Do you have hypotension?	8	−1
Do you have complications of cardiac and vascular prosthetic devices, implants and grafts?	6	0
Are you vitamin D deficient?	2	0
Do you have cardiac arrhythmias?	2	0

## Discussion

In this project, we built a data processing and predictive analytics workflow to predict the risks for COVID-19 diagnosis using patients’ longitudinal medical conditions encoded by the ICD-9/ICD-10 classification system. We tested the implications of applying different time windows and alternative variable regularization methods to extract the most predictive features from the condition data.

Although the all-time data model selected more features with implications about pre-existing chronic medical conditions increasing the risk of contracting COVID-19, we determined that it was prone to capturing spurious correlations with distant historical data and had weaker performance than the 2-week models (Figures 2, 3; Table 2; Supplemental Tables S1–S4). With regards to modeling techniques, we found that a more stringent regularized regression approach such as LASSO resulted in simpler models and still achieved high performance as compared to more complex models built from the Elastic-Net method (Figures 2, 3; Table 2; Supplemental Tables S1–S4). As simpler models tend to be more generalizable, more interpretable, and less likely to be overfit, we consider the LASSO model using the 2-week data filter the superior model for its parsimony without sacrificing performance. Many COVID-19 risk prediction studies also employed LASSO (Alballa and Al-Turaiki, 2021) with a few other studies used Elastic-Net (Heldt et al., 2021; Hu et al., 2021; Huang et al., 2021) as feature selection methods. A COVID-19 diagnostic prediction study by (Feng et al., 2021) compared the performance of four different feature selection methods including LASSO, Ridge, Decision Tree and AdaBoost also found LASSO produced the best performance in both the testing and the validation set.

While our workflow focuses on automatically extracting predictive features from ICD9/10 codes, the majority of COVID-19 prediction studies selected features from a wide-range of additional clinical data components such as chest computed tomography (CT) scan results, laboratory blood tests, which includes complete blood count (e.g., leukocyte, erythrocyte, platelet count, and hematocrit), metabolic factors (e.g., glucose, sodium, potassium, creatinine, urea, albumin, and bilirubin), clotting factors (e.g., prothrombin and fibrinogen), inflammation markers such as C-reactive protein and interleukin 6 (IL-6) (Alballa and Al-Turaiki, 2021). Furthermore, whereas some studies selected the initial sets of features from EHR data based on expert opinions (Estiri et al., 2021; Feng et al., 2021; Schwab et al., 2021) and/or literature review (Joshi et al., 2020; Schwab et al., 2021), we took an unbiased approach to use ICD9/10 codes along with demographic information as the initial set of features. Our data wrangling workflow is limited to the data available in the OMOP common data model, which facilitates scaling up the analyses when we have access to more data of the same format in the future.

Our results showed several COVID-19 predictive features that overlapped with existing published findings. For example, several respiratory symptoms such as cough, abnormalities of breath, and chest pain prioritized by our models—particularly within the 2-week timeframe—are well-known symptoms of COVID-19 (Fu et al., 2020; Huang et al., 2020). Other chronic conditions selected from our models have also been reported to increase COVID-19 risks such as obesity (Popkin et al., 2020), allergic rhinitis (Yang et al., 2020), cardiovascular diseases (Nishiga et al., 2020) and kidney diseases (Adapa et al., 2020) while there are still on-going debates about the role of nicotine and smoking in COVID-19 risks (Polosa and Caci, 2020). Similar to other studies, we found that major depressive disorder is associated with COVID-19 diagnoses. However, it is unclear whether severe mental health problems are the cause, the effect, or the confounding factors with other features associated with COVID-19 (Ettman et al., 2020; Nami et al., 2020; Skoda et al., 2020).

A major limitation in our predictive modeling pipeline relates to the fact that our model is based entirely on correlations between medical conditions and COVID-19 testing/diagnosis. Therefore, by design, this workflow cannot establish causal relationships. As examples, there are several medical conditions associated with lower risks for COVID-19 (Supplemental Tables S1–S4) which may highlight distinct features in our negative cohort but may not directly affect COVID-19 risks. This problem, however, is inevitable in predictive analytic workflows that derive inferences from retrospective data. Similar to all studies that apply machine learning methods to model COVID-19 diagnosis, our classifier is prone to imbalanced class distribution where there the positive COVID-19 instances are underrepresented in the training data (Alballa and Al-Turaiki, 2021). However, we addressed this class imbalance issue by weighing each observation inversely proportional to the size of its class (see the Methods *Variable (Feature) Selections*). Finally; we choose a generalized linear model approach where we assume linear relationships on a logistic scale between medical conditions and COVID-19 risks, and consequently, potential non-linear relationships are not considered.

Although our workflow is straightforward to implement, there are substantial trade-offs by using the ICD-9/ICD-10 standard vocabulary system as opposed to alternative text mining approaches to extract medical conditions from EHR data. ICD code accuracy is a major problem in some cases with classification error rates as high as 80% (O'Malley et al., 2005). The sources of these errors are wide-ranging including poor communication between patients and providers, clinician’ mistakes or biases, transcription/scanning errors, coders’ experience, and intentional or unintentional biases (e.g., upcoding and unbundling for higher billing/reimbursement value) (O'Malley et al., 2005). Inconsistent, incomplete, systemic and random errors in ICD coding (Cox et al., 2009) introduce noise in the dataset, which is another limitation of our workflow.

Despite these inherent limitations, our study shows the promising utility of incorporating the ICD-10 system in an unbiased manner for novel inferences of EHR data, particularly to study medical symptoms and conditions that influence the risks for COVID-19. Future studies can consider incorporating other standard vocabularies available in EHR data such as Systemized Nomenclature of Medicine (SNOMED), Current Procedural Terminology (CPT), Logical Observation Identifiers Names and Codes (LOINC) as well as adding additional datasets such as patient’ medication uses to further understand the risks and the long-term consequences of COVID-19.

## Data Availability

The data analyzed in this study is subject to the following licenses/restrictions: All restrictions of the Limited Data Set (LDS) from the UAB i2b2 system apply to this dataset. Requests to access these datasets should be directed to https://www.uab.edu/ccts/research-commons/berd/55-research-commons/informatics/325-i2b2.
