# Supplementary material for: Development of An Individualized Risk Prediction Model for COVID-19 Using Electronic Health Record Data
Source: Front Big Data. 2021 Jun 4;4:675882. doi: 10.3389/fdata.2021.675882 (PMC8211871; doi:10.3389/fdata.2021.675882)
Supplement: Supplementary file 1 [file datasheet1.docx]

Supplementary Material

**Supplementary Figure 1 | Score Distribution.** COVID-19 risk score distribution of the model based on the LASSO method using 2-week data filter. Scores are calculated from the coefficients and WoE transformations of the logistic regression model. We used the default settings in the scorecardpy package, which set the target odds to 1/19, the corresponding target points to 600, and the default points required to double the odds to 50. The left y-axis and bars indicate the prevalence of scores in the samples. The right y-axis and line plots indicate the probability of not having a COVID-19 diagnosis (COVID-19 negative). (Note: Credit Scorecard models are typically used to indicate higher credit scores are more favorable. For COVID-19 risks, the terminology is reversed such that higher risk scores are worse as indicated by the “bad probability” annotation on the right axis.) The Population Stability Index (PSI) in the upper right of each panel compares the distribution of scores across the training and testing data set (Yurdakul and Naranjo, 2019). A score of 0.1 or less indicates that distributions are reasonably similar between the two sets.

**Supplementary Table 1 | Scorecard Derived from 2-Week LASSO Model**

For converting the coefficients and the WoE transformations of the logistic regression model to scores, we used the package defaults from scorecardpy, which set the target odds to 1/19, the corresponding target points to 600, and the default points required to double the odds to 50. The resulting point values for each feature are shown here. A positive score indicates that a feature pushes a prediction toward positive COVID-19 test/diagnosis.

| Variables | COVID-19 diagnosis | Points  (base points = 320) | ICD-10 annotation |
| --- | --- | --- | --- |
| AGE (40.0, 50.0] | negative | -3 |  |
| AGE (40.0, 50.0] | positive | 19 |  |
| AGE (65.0, 75.0] | negative | 3 |  |
| AGE (65.0, 75.0] | positive | -20 |  |
| BMI-20.0_24.9 | negative | 2 |  |
| BMI-20.0_24.9 | positive | -27 |  |
| BMI-unknown | negative | -11 |  |
| BMI-unknown | positive | 126 |  |
| current_smoker | negative | 7 |  |
| current_smoker | positive | -42 |  |
| current_Substance_use | negative | 9 |  |
| current_Substance_use | positive | -123 |  |
| E66 | negative | -1 | Overweight and Obesity |
| E66 | positive | 12 | Overweight and Obesity |
| E87 | negative | -5 | Other disorders of fluid, electrolyte and acid-base balance |
| E87 | positive | 48 | Other disorders of fluid, electrolyte and acid-base balance |
| F17 | negative | -1 | nicotine dependence |
| F17 | positive | 15 | nicotine dependence |
| F32 | negative | -2 | Major Depressive Disorder |
| F32 | positive | 24 | Major Depressive Disorder |
| former_alcohol | negative | 4 |  |
| former_alcohol | positive | -47 |  |
| former_smoker | negative | 4 |  |
| former_smoker | positive | -13 |  |
| former_Substance_use | negative | 3 |  |
| former_Substance_use | positive | -53 |  |
| I25 | negative | 2 | Chronic Ischemic Heart Disease |
| I25 | positive | -25 | Chronic Ischemic Heart Disease |
| J98 | negative | 0 | Respiratory disorder, unspecified |
| J98 | positive | 7 | Respiratory disorder, unspecified |
| M | negative | 5 |  |
| M | positive | -6 |  |
| N18 | negative | 2 | Chronic kidney disease |
| N18 | positive | -31 | Chronic kidney disease |
| No_alcohol | negative | 10 |  |
| No_alcohol | positive | -11 |  |
| No_Substance_use | negative | 13 |  |
| No_Substance_use | positive | -5 |  |
| O99 | negative | 0 | Diseases of the circulatory system complicating pregnancy, childbirth and the puerperium |
| O99 | positive | -9 | Diseases of the circulatory system complicating pregnancy, childbirth and the puerperium |
| R05 | negative | -45 | cough |
| R05 | positive | 60 | cough |
| R06 | negative | -7 | Abnormalities of breathing |
| R06 | positive | 42 | Abnormalities of breathing |
| R07 | negative | -3 | Pain in throat and chest. |
| R07 | positive | 26 | Pain in throat and chest. |
| R10 | negative | 2 | Abdominal and pelvic pain |
| R10 | positive | -43 | Abdominal and pelvic pain |
| R45 | negative | 2 | Symptoms and signs involving emotional state |
| R45 | positive | -94 | Symptoms and signs involving emotional state |
| R91 | negative | -4 | Abnormal findings on diagnostic imaging of lung |
| R91 | positive | 43 | Abnormal findings on diagnostic imaging of lung |
| Unknown | negative | -3 | Unknown ethnicity |
| Unknown | positive | 29 | Unknown ethnicity |
| unknown_alcohol | negative | 6 |  |
| unknown_alcohol | positive | -56 |  |
| White | negative | 21 |  |
| White | positive | -26 |  |
| Z3A | negative | 7 | pregnancy |
| Z3A | positive | -93 | pregnancy |

**Supplementary Table 2 | Scorecard Derived from All Time LASSO Model**

For converting the coefficients and the WoE transformations of the logistic regression model to scores, we used the package defaults from scorecardpy, which set the target odds to 1/19, the corresponding target points to 600, and the default points required to double the odds to 50. The resulting point values for each feature are shown here. A positive score indicates that a feature pushes a prediction toward positive COVID-19 test/diagnosis.

| Variables | COVID-19 diagnosis | Points  (base points = 319) | ICD-10 annotation |
| --- | --- | --- | --- |
| AGE_(40.0, 50.0] | negative | -4 |  |
| AGE_(40.0, 50.0] | positive | 20 |  |
| AGE_(65.0, 75.0] | negative | 2 |  |
| AGE_(65.0, 75.0] | positive | -12 |  |
| B95 | negative | 1 | Streptococcus, Staphylococcus, and Enterococcus |
| B95 | positive | -12 | Streptococcus, Staphylococcus, and Enterococcus |
| B96 | negative | -1 | Other bacterial agents as the cause of diseases classified elsewhere |
| B96 | positive | 9 | Other bacterial agents as the cause of diseases classified elsewhere |
| BMI-20.0_24.9 | negative | 1 |  |
| BMI-20.0_24.9 | positive | -15 |  |
| current_smoker | negative | 7 |  |
| current_smoker | positive | -39 |  |
| current_Substance_use | negative | 9 |  |
| current_Substance_use | positive | -110 |  |
| D62 | negative | 1 | Acute posthemorrhagic anemia |
| D62 | positive | -9 | Acute posthemorrhagic anemia |
| D63 | negative | -1 | Anemia |
| D63 | positive | 10 | Anemia |
| D72 | negative | 2 | Elevated white blood cell count |
| D72 | positive | -12 | Elevated white blood cell count |
| E55 | negative | 0 | Vitamin D deficiency |
| E55 | positive | 2 | Vitamin D deficiency |
| E83 | negative | 2 | Disorder of iron metabolism, unspecified. |
| E83 | positive | -9 | Disorder of iron metabolism, unspecified. |
| F11 | negative | 1 | opioid related disorders |
| F11 | positive | -13 | opioid related disorders |
| F12 | negative | 3 | Substance use disorder |
| F12 | positive | -66 | Substance use disorder |
| F14 | negative | 0 | Cocaine related disorders |
| F14 | positive | -9 | Cocaine related disorders |
| F17 | negative | -4 | nicotine dependence |
| F17 | positive | 14 | nicotine dependence |
| F29 | negative | 2 | Unspecified psychosis not due to a substance or known physiological condition |
| F29 | positive | -60 | Unspecified psychosis not due to a substance or known physiological condition |
| F31 | negative | 1 | Bipolar disorder |
| F31 | positive | -30 | Bipolar disorder |
| F32 | negative | 0 | Major Depressive Disorder |
| F32 | positive | 1 | Major Depressive Disorder |
| F41 | negative | -2 | Generalized Anxiety Disorder |
| F41 | positive | 5 | Generalized Anxiety Disorder |
| F43 | negative | 1 | Post-Traumatic Stress Disorder |
| F43 | positive | -17 | Post-Traumatic Stress Disorder |
| former_alcohol | negative | 3 |  |
| former_alcohol | positive | -29 |  |
| former_smoker | negative | 0 |  |
| former_smoker | positive | -1 |  |
| former_Substance_use | negative | 5 |  |
| former_Substance_use | positive | -93 |  |
| G31 | negative | 1 | Other degenerative diseases of nervous system, not elsewhere classified. |
| G31 | positive | -23 | Other degenerative diseases of nervous system, not elsewhere classified. |
| G62 | negative | 1 | Polyneuropathy |
| G62 | positive | -8 | Polyneuropathy |
| G93 | negative | -1 | Other disorders of brain. |
| G93 | positive | 7 | Other disorders of brain. |
| I12 | negative | -1 | Hypertensive chronic kidney disease with stage 5 chronic kidney disease or end stage renal disease. |
| I12 | positive | 8 | Hypertensive chronic kidney disease with stage 5 chronic kidney disease or end stage renal disease. |
| I13 | negative | -1 | Hypertensive heart and chronic kidney disease with heart failure and stage 1 through stage 4 chronic kidney disease, or unspecified chronic kidney disease |
| I13 | positive | 13 | Hypertensive heart and chronic kidney disease with heart failure and stage 1 through stage 4 chronic kidney disease, or unspecified chronic kidney disease |
| I25 | negative | 2 | Chronic Ischemic Heart Disease |
| I25 | positive | -11 | Chronic Ischemic Heart Disease |
| I34 | negative | 0 | Nonrheumatic mitral (valve) insufficiency |
| I34 | positive | -1 | Nonrheumatic mitral (valve) insufficiency |
| I48 | negative | 0 | Atrial fibrillation and flutter. |
| I48 | positive | -4 | Atrial fibrillation and flutter. |
| I49 | negative | 0 | Other cardiac arrhythmias. |
| I49 | positive | 2 | Other cardiac arrhythmias. |
| I51 | negative | -2 | Complications and ill-defined descriptions of heart disease. |
| I51 | positive | 8 | Complications and ill-defined descriptions of heart disease. |
| I82 | negative | 0 | Other venous embolism and thrombosis. |
| I82 | positive | -2 | Other venous embolism and thrombosis. |
| I95 | negative | -1 | Hypotension. |
| I95 | positive | 8 | Hypotension. |
| J06 | negative | -6 | Acute upper respiratory infections of multiple and unspecified sites. |
| J06 | positive | 30 | Acute upper respiratory infections of multiple and unspecified sites. |
| J18 | negative | -3 | Pneumonia, unspecified organism. |
| J18 | positive | 17 | Pneumonia, unspecified organism. |
| J30 | negative | -2 | Vasomotor and allergic rhinitis. |
| J30 | positive | 14 | Vasomotor and allergic rhinitis. |
| J34 | negative | 0 | Other and unspecified disorders of nose and nasal sinuses. |
| J34 | positive | 4 | Other and unspecified disorders of nose and nasal sinuses. |
| J90 | negative | 1 | pleural effusion |
| J90 | positive | -11 | pleural effusion |
| J96 | negative | -1 | Respiratory failure |
| J96 | positive | 5 | Respiratory failure |
| J98 | negative | -2 | Respiratory disorder, unspecified |
| J98 | positive | 7 | Respiratory disorder, unspecified |
| K31 | negative | 0 | Other diseases of stomach and duodenum. |
| K31 | positive | -8 | Other diseases of stomach and duodenum. |
| K42 | negative | 2 | Umbilical hernia with obstruction |
| K42 | positive | -38 | Umbilical hernia with obstruction |
| K44 | negative | -1 | Diaphragmatic hernia with obstruction, without gangrene |
| K44 | positive | 13 | Diaphragmatic hernia with obstruction, without gangrene |
| K52 | negative | 1 | Other noninfective gastroenteritis and colitis. |
| K52 | positive | -8 | Other noninfective gastroenteritis and colitis. |
| K59 | negative | 1 | Other functional intestinal disorders. |
| K59 | positive | -4 | Other functional intestinal disorders. |
| K74 | negative | -1 | Fibrosis and cirrhosis of liver. |
| K74 | positive | 13 | Fibrosis and cirrhosis of liver. |
| K86 | negative | 3 | Other diseases of pancreas. |
| K86 | positive | -66 | Other diseases of pancreas. |
| M48 | negative | 1 | Spinal stenosis |
| M48 | positive | -13 | Spinal stenosis |
| M89 | negative | 2 | Disorder of bone |
| M89 | positive | -24 | Disorder of bone |
| N17 | negative | -4 | Acute kidney failure |
| N17 | positive | 15 | Acute kidney failure |
| N18 | negative | 8 | Chronic kidney disease |
| N18 | positive | -41 | Chronic kidney disease |
| N20 | negative | 3 | Calculus of kidney and ureter. |
| N20 | positive | -38 | Calculus of kidney and ureter. |
| N28 | negative | 2 | Other disorders of kidney and ureter, not elsewhere classified. |
| N28 | positive | -11 | Other disorders of kidney and ureter, not elsewhere classified. |
| N32 | negative | 1 | Other disorders of bladder. |
| N32 | positive | -16 | Other disorders of bladder. |
| No_alcohol | negative | 7 |  |
| No_alcohol | positive | -7 |  |
| No_Substance_use | negative | 34 |  |
| No_Substance_use | positive | -13 |  |
| O09 | negative | 3 | supervision of high risk pregnancy. |
| O09 | positive | -43 | supervision of high risk pregnancy. |
| R00 | negative | 0 | Abnormalities of heart beat. |
| R00 | positive | -1 | Abnormalities of heart beat. |
| R05 | negative | -44 | cough |
| R05 | positive | 36 | cough |
| R10 | negative | 0 | abdominal and pelvic pain |
| R10 | positive | -1 | abdominal and pelvic pain |
| R11 | negative | 1 | Nausea and vomiting |
| R11 | positive | -4 | Nausea and vomiting |
| R16 | negative | 1 | Hepatomegaly and splenomegaly |
| R16 | positive | -7 | Hepatomegaly and splenomegaly |
| R18 | negative | 1 | Ascites. |
| R18 | positive | -19 | Ascites. |
| R20 | negative | -2 | Disturbances of skin sensation. |
| R20 | positive | 12 | Disturbances of skin sensation. |
| R21 | negative | 1 | Rash and other nonspecific skin eruption |
| R21 | positive | -7 | Rash and other nonspecific skin eruption |
| R33 | negative | -1 | Retention of urine. |
| R33 | positive | 13 | Retention of urine. |
| R35 | negative | 0 | polyurea |
| R35 | positive | 1 | polyurea |
| R41 | negative | -1 | Other symptoms and signs involving cognitive functions and awareness |
| R41 | positive | 4 | Other symptoms and signs involving cognitive functions and awareness |
| R45 | negative | 1 | Symptoms and signs involving emotional state |
| R45 | positive | -18 | Symptoms and signs involving emotional state |
| R47 | negative | 1 | Unspecified speech disturbances |
| R47 | positive | -27 | Unspecified speech disturbances |
| R50 | negative | -5 | fever |
| R50 | positive | 24 | fever |
| R52 | negative | -1 | pain |
| R52 | positive | 14 | pain |
| R59 | negative | 2 | Localized enlarged lymph nodes |
| R59 | positive | -13 | Localized enlarged lymph nodes |
| R79 | negative | 3 | Other abnormal findings of blood chemistry. |
| R79 | positive | -20 | Other abnormal findings of blood chemistry. |
| R93 | negative | 1 | Abnormal findings on diagnostic imaging of other specified body structures |
| R93 | positive | -7 | Abnormal findings on diagnostic imaging of other specified body structures |
| S00 | negative | 1 | Superficial injury of head. |
| S00 | positive | -14 | Superficial injury of head. |
| S09 | negative | 2 | Other and unspecified injuries of head |
| S09 | positive | -29 | Other and unspecified injuries of head |
| T14 | negative | 1 | injury |
| T14 | positive | -13 | injury |
| T82 | negative | 0 | Complications of cardiac and vascular prosthetic devices, implants and grafts. |
| T82 | positive | 6 | Complications of cardiac and vascular prosthetic devices, implants and grafts. |
| Unknown | negative | -5 | Unknown ethnicity |
| Unknown | positive | 49 | Unknown ethnicity |
| White | negative | 22 |  |
| White | positive | -27 |  |
| Z37 | negative | -2 | Outcome of delivery |
| Z37 | positive | 35 | Outcome of delivery |
| Z3A | negative | 6 | pregnancy |
| Z3A | positive | -62 | pregnancy |
| Z78 | negative | 1 | Other long term (current) drug therapy |
| Z78 | positive | -13 | Other long term (current) drug therapy |
| Z90 | negative | 2 | Acquired absence of organs, not elsewhere classified. |
| Z90 | positive | -10 | Acquired absence of organs, not elsewhere classified. |
| Z95 | negative | 1 | Presence of cardiac and vascular implants and grafts |
| Z95 | positive | -14 | Presence of cardiac and vascular implants and grafts |
| Z99 | negative | 1 | Dependence on enabling machines and devices |
| Z99 | positive | -14 | Dependence on enabling machines and devices |

**Supplementary Table 3 | Scorecard Derived from 2-week Elastic-Net Model**

For converting the coefficients and the WoE transformations of the logistic regression model to scores, we used the package defaults from scorecardpy, which set the target odds to 1/19, the corresponding target points to 600, and the default points required to double the odds to 50. The resulting point values for each feature are shown here. A positive score indicates that a feature pushes a prediction toward positive COVID-19 test/diagnosis.

| Variables | COVID-19 diagnosis | Points  (base points = 390) | ICD-10 annotation |
| --- | --- | --- | --- |
| AGE_(18.0, 30.0] | negative | -1 |  |
| AGE_(18.0, 30.0] | positive | 3 |  |
| AGE_(40.0, 50.0] | negative | -4 |  |
| AGE_(40.0, 50.0] | positive | 20 |  |
| AGE_(50.0, 65.0] | negative | -1 |  |
| AGE_(50.0, 65.0] | positive | 2 |  |
| AGE_(65.0, 75.0] | negative | 3 |  |
| AGE_(65.0, 75.0] | positive | -18 |  |
| Black or African American | negative | 1 |  |
| Black or African American | positive | -2 |  |
| BMI-20.0_24.9 | negative | 2 |  |
| BMI-20.0_24.9 | positive | -28 |  |
| current_smoker | negative | 7 |  |
| current_smoker | positive | -43 |  |
| current_Substance_use | negative | 8 |  |
| current_Substance_use | positive | -113 |  |
| E66 | negative | -1 | Overweight and Obesity |
| E66 | positive | 9 | Overweight and Obesity |
| E78 | negative | -1 | Hyperlipidemia, Unspecified |
| E78 | positive | 4 | Hyperlipidemia, Unspecified |
| E87 | negative | -3 | Other disorders of fluid, electrolyte and acid-base balance |
| E87 | positive | 36 | Other disorders of fluid, electrolyte and acid-base balance |
| F17 | negative | -1 | Nicotine dependence |
| F17 | positive | 17 | Nicotine dependence |
| F32 | negative | -2 | Major Depressive Disorder |
| F32 | positive | 23 | Major Depressive Disorder |
| F41 | negative | 0 | Generalized Anxiety Disorder |
| F41 | positive | -2 | Generalized Anxiety Disorder |
| Female | negative | -3 |  |
| Female | positive | 2 |  |
| former_alcohol | negative | 4 |  |
| former_alcohol | positive | -44 |  |
| former_smoker | negative | 4 |  |
| former_smoker | positive | -13 |  |
| former_Substance_use | negative | 2 |  |
| former_Substance_use | positive | -43 |  |
| I10 | negative | 0 | Essential (Primary) Hypertension |
| I10 | positive | 1 | Essential (Primary) Hypertension |
| I25 | negative | 2 | Chronic Ischemic Heart Disease |
| I25 | positive | -24 | Chronic Ischemic Heart Disease |
| J98 | negative | -1 | Other disorders of lung |
| J98 | positive | 14 | Other disorders of lung |
| K21 | negative | 0 | Gastro-esophageal reflux disease with esophagitis |
| K21 | positive | 1 | Gastro-esophageal reflux disease with esophagitis |
| M | negative | 3 |  |
| M | positive | -4 |  |
| M79 | negative | 0 | Other and unspecified soft tissue disorders |
| M79 | positive | -1 | Other and unspecified soft tissue disorders |
| N18 | negative | 2 | Chronic kidney disease |
| N18 | positive | -23 | Chronic kidney disease |
| never_smoker | negative | 1 |  |
| never_smoker | positive | -1 |  |
| No_alcohol | negative | 10 |  |
| No_alcohol | positive | -11 |  |
| No_Substance_use | negative | 8 |  |
| No_Substance_use | positive | -3 |  |
| Non-Hispanic/Latino | negative | 21 |  |
| Non-Hispanic/Latino | positive | -3 |  |
| O09 | negative | 0 | Supervision of high risk pregnancy |
| O09 | positive | -2 | Supervision of high risk pregnancy |
| O99 | negative | 1 | Other maternal diseases classifiable elsewhere but complicating pregnancy, childbirth and the puerperium |
| O99 | positive | -15 | Other maternal diseases classifiable elsewhere but complicating pregnancy, childbirth and the puerperium |
| R00 | negative | 0 | Abnormalities of heart beat. |
| R00 | positive | -1 | Abnormalities of heart beat. |
| R05 | negative | -45 | Cough |
| R05 | positive | 60 | Cough |
| R06 | negative | -7 | Abnormalities of breathing |
| R06 | positive | 42 | Abnormalities of breathing |
| R07 | negative | -2 | Pain in throat and chest |
| R07 | positive | 21 | Pain in throat and chest |
| R10 | negative | 2 | Abdominal and pelvic pain |
| R10 | positive | -35 | Abdominal and pelvic pain |
| R45 | negative | 2 | Symptoms and signs involving emotional status |
| R45 | positive | -94 | Symptoms and signs involving emotional status |
| R91 | negative | -4 | Abnormal findings on diagnostic imaging of lung |
| R91 | positive | 38 | Abnormal findings on diagnostic imaging of lung |
| unknown_alcohol | negative | 2 |  |
| unknown_alcohol | positive | -20 |  |
| unknown_smoker | negative | -6 |  |
| unknown_smoker | positive | 62 |  |
| unknown_Substance_use | negative | -3 |  |
| unknown_Substance_use | positive | 28 |  |
| White | negative | 22 |  |
| White | positive | -26 |  |
| Z3A | negative | 7 | Pregnancy |
| Z3A | positive | -93 | Pregnancy |

**Supplementary Table 4 | Scorecard Derived from All Time Elastic-Net Model**

For converting the coefficients and the WoE transformations of the logistic regression model to scores, we used the package defaults from scorecardpy, which set the target odds to 1/19, the corresponding target points to 600, and the default points required to double the odds to 50. The resulting point values for each feature are shown here. A positive score indicates that a feature pushes a prediction toward positive COVID-19 test/diagnosis.

| Variables | COVID-19 diagnosis | Points  (base points = 388) | ICD-10 annotation |
| --- | --- | --- | --- |
| A41 | negative | 0 | sepsis |
| A41 | positive | 2 | sepsis |
| AGE_(30.0, 40.0] | negative | 0 |  |
| AGE_(30.0, 40.0] | positive | 2 |  |
| AGE_(40.0, 50.0] | negative | -3 |  |
| AGE_(40.0, 50.0] | positive | 17 |  |
| AGE_(50.0, 65.0] | negative | 0 |  |
| AGE_(50.0, 65.0] | positive | -1 |  |
| AGE_(65.0, 75.0] | negative | 2 |  |
| AGE_(65.0, 75.0] | positive | -11 |  |
| B19 | negative | 0 | Unspecified viral hepatitis |
| B19 | positive | 4 | Unspecified viral hepatitis |
| B35 | negative | 0 | Dermatophytosis |
| B35 | positive | 1 | Dermatophytosis |
| B95 | negative | 1 | Streptococcus, Staphylococcus, and Enterococcus |
| B95 | positive | -12 | Streptococcus, Staphylococcus, and Enterococcus |
| B96 | negative | -1 | Other bacterial agents as the cause of diseases classified elsewhere |
| B96 | positive | 11 | Other bacterial agents as the cause of diseases classified elsewhere |
| BMI-20.0_24.9 | negative | 1 |  |
| BMI-20.0_24.9 | positive | -18 |  |
| BMI-25.0_39.9 | negative | 1 |  |
| BMI-25.0_39.9 | positive | -1 |  |
| current_smoker | negative | 8 |  |
| current_smoker | positive | -43 |  |
| current_Substance_use | negative | 7 |  |
| current_Substance_use | positive | -85 |  |
| D12 | negative | 0 | Benign neoplasm of colon, rectum, anus and anal canal |
| D12 | positive | 2 | Benign neoplasm of colon, rectum, anus and anal canal |
| D50 | negative | 0 | Iron deficiency anemia secondary to blood loss |
| D50 | positive | 3 | Iron deficiency anemia secondary to blood loss |
| D62 | negative | 1 | Acute blood loss anemia |
| D62 | positive | -10 | Acute blood loss anemia |
| D63 | negative | -1 | Anemia in chronic diseases |
| D63 | positive | 11 | Anemia in chronic diseases |
| D64 | negative | 1 | Anemia, Unspecified |
| D64 | positive | -3 | Anemia, Unspecified |
| D69 | negative | 0 | Thrombocytopenia |
| D69 | positive | 4 | Thrombocytopenia |
| D72 | negative | 3 | Elevated white blood cell count |
| D72 | positive | -13 | Elevated white blood cell count |
| E03 | negative | 0 | Other hypothyroidism |
| E03 | positive | -2 | Other hypothyroidism |
| E55 | negative | -1 | Vitamin D deficiency |
| E55 | positive | 5 | Vitamin D deficiency |
| E66 | negative | 0 | Overweight and Obesity |
| E66 | positive | 1 | Overweight and Obesity |
| E83 | negative | 2 | Disorder of iron metabolism |
| E83 | positive | -10 | Disorder of iron metabolism |
| E86 | negative | -1 | Volume depletion |
| E86 | positive | 6 | Volume depletion |
| E87 | negative | 1 | Other disorders of fluid, electrolyte and acid-base balance |
| E87 | positive | -1 | Other disorders of fluid, electrolyte and acid-base balance |
| E88 | negative | 0 | Metabolic disorder |
| E88 | positive | 1 | Metabolic disorder |
| F | negative | -1 |  |
| F | positive | 1 |  |
| F10 | negative | 0 | Mental and behavioural disorders due to use of alcohol |
| F10 | positive | -6 | Mental and behavioural disorders due to use of alcohol |
| F11 | negative | 1 | Opioid related disorders |
| F11 | positive | -20 | Opioid related disorders |
| F12 | negative | 3 | Cannabis related disorders |
| F12 | positive | -62 | Cannabis related disorders |
| F14 | negative | 1 | Cocain related disorders |
| F14 | positive | -16 | Cocain related disorders |
| F17 | negative | -5 | Nicotine dependence |
| F17 | positive | 17 | Nicotine dependence |
| F19 | negative | -1 | Other psychoactive substance related disorders |
| F19 | positive | 9 | Other psychoactive substance related disorders |
| F29 | negative | 2 | Unspecified psychosis not due to a substance or known physiological condition |
| F29 | positive | -57 | Unspecified psychosis not due to a substance or known physiological condition |
| F31 | negative | 1 | Bipolar disorder |
| F31 | positive | -30 | Bipolar disorder |
| F32 | negative | -1 | Major Depressive Disorder |
| F32 | positive | 2 | Major Depressive Disorder |
| F41 | negative | -2 | Generalized Anxiety Disorder |
| F41 | positive | 7 | Generalized Anxiety Disorder |
| F43 | negative | 2 | Post-Traumatic Stress Disorder |
| F43 | positive | -18 | Post-Traumatic Stress Disorder |
| former_alcohol | negative | 3 |  |
| former_alcohol | positive | -30 |  |
| former_smoker | negative | 2 |  |
| former_smoker | positive | -5 |  |
| former_Substance_use | negative | 3 |  |
| former_Substance_use | positive | -60 |  |
| G31 | negative | 1 | Other degenerative diseases of nervous system, not elsewhere classified |
| G31 | positive | -23 | Other degenerative diseases of nervous system, not elsewhere classified |
| G40 | negative | 0 | Epilepsy and recurrent seizures |
| G40 | positive | 5 | Epilepsy and recurrent seizures |
| G43 | negative | 0 | Migraine |
| G43 | positive | 1 | Migraine |
| G47 | negative | 1 | Insomnia |
| G47 | positive | -3 | Insomnia |
| G62 | negative | 1 | Polyneuropathy |
| G62 | positive | -11 | Polyneuropathy |
| G89 | negative | 0 | Pain |
| G89 | positive | -1 | Pain |
| G93 | negative | -2 | Other disorders of brain |
| G93 | positive | 8 | Other disorders of brain |
| I10 | negative | -1 | Essential (Primary) Hypertension |
| I10 | positive | 1 | Essential (Primary) Hypertension |
| I11 | negative | 0 | Hypertensive heart disease with heart failure |
| I11 | positive | -3 | Hypertensive heart disease with heart failure |
| I12 | negative | -1 | Hypertensive chronic kidney disease with stage 5 chronic kidney disease or end stage renal disease |
| I12 | positive | 7 | Hypertensive chronic kidney disease with stage 5 chronic kidney disease or end stage renal disease |
| I13 | negative | -1 | Hypertensive heart and chronic kidney disease with heart failure and stage 1 through stage 4 chronic kidney disease, or unspecified chronic kidney disease |
| I13 | positive | 11 | Hypertensive heart and chronic kidney disease with heart failure and stage 1 through stage 4 chronic kidney disease, or unspecified chronic kidney disease |
| I16 | negative | 0 | Hypertensive Crisis |
| I16 | positive | -1 | Hypertensive Crisis |
| I21 | negative | 0 | Acute myocardial infarction |
| I21 | positive | 1 | Acute myocardial infarction |
| I25 | negative | 3 | Chronic Ischemic Heart Disease |
| I25 | positive | -12 | Chronic Ischemic Heart Disease |
| I27 | negative | 0 | Other pulmonary heart diseases |
| I27 | positive | 5 | Other pulmonary heart diseases |
| I34 | negative | 1 | Nonrheumatic mitral (valve) insufficiency |
| I34 | positive | -9 | Nonrheumatic mitral (valve) insufficiency |
| I42 | negative | 0 | Cardiomyopathy |
| I42 | positive | 9 | Cardiomyopathy |
| I47 | negative | 0 | Supraventricular tachycardia |
| I47 | positive | -2 | Supraventricular tachycardia |
| I48 | negative | 1 | Atrial fibrillation and flutter |
| I48 | positive | -9 | Atrial fibrillation and flutter |
| I49 | negative | -1 | Other cardiac arrhythmias |
| I49 | positive | 6 | Other cardiac arrhythmias |
| I50 | negative | 1 | Acute decompensated heart failure |
| I50 | positive | -3 | Acute decompensated heart failure |
| I51 | negative | -1 | Complications and ill-defined descriptions of heart disease |
| I51 | positive | 6 | Complications and ill-defined descriptions of heart disease |
| I63 | negative | 0 | Cerebral infarction |
| I63 | positive | 2 | Cerebral infarction |
| I65 | negative | 0 | Occlusion and stenosis of precerebral arteries, not resulting in cerebral infarction |
| I65 | positive | -3 | Occlusion and stenosis of precerebral arteries, not resulting in cerebral infarction |
| I67 | negative | 0 | Other cerebrovascular diseases |
| I67 | positive | 1 | Other cerebrovascular diseases |
| I70 | negative | 0 | Unspecified atherosclerosis of native arteries of extremities, right leg |
| I70 | positive | 2 | Unspecified atherosclerosis of native arteries of extremities, right leg |
| I73 | negative | 0 | Peripheral vascular disease |
| I73 | positive | 6 | Peripheral vascular disease |
| I82 | negative | 1 | Other venous embolism and thrombosis |
| I82 | positive | -6 | Other venous embolism and thrombosis |
| I95 | negative | -1 | Hypotension |
| I95 | positive | 9 | Hypotension |
| J02 | negative | 0 | Acute pharyngitis |
| J02 | positive | 2 | Acute pharyngitis |
| J06 | negative | -5 | Acute upper respiratory infections of multiple and unspecified sites |
| J06 | positive | 26 | Acute upper respiratory infections of multiple and unspecified sites |
| J18 | negative | -1 | Pneumonia, unspecified organism |
| J18 | positive | 5 | Pneumonia, unspecified organism |
| J20 | negative | 0 | Acute Bronchitis |
| J20 | positive | 4 | Acute Bronchitis |
| J30 | negative | -2 | Vasomotor and allergic rhinitis |
| J30 | positive | 12 | Vasomotor and allergic rhinitis |
| J32 | negative | 0 | Chronic sinusitis |
| J32 | positive | 1 | Chronic sinusitis |
| J34 | negative | 0 | Other and unspecified disorders of nose and nasal sinuses |
| J34 | positive | 6 | Other and unspecified disorders of nose and nasal sinuses |
| J43 | negative | 0 | Emphysema |
| J43 | positive | 1 | Emphysema |
| J44 | negative | 0 | Chronic obstructive pulmonary disease |
| J44 | positive | -1 | Chronic obstructive pulmonary disease |
| J45 | negative | 0 | Asthma |
| J45 | positive | 2 | Asthma |
| J81 | negative | -1 | Pulmonary edema |
| J81 | positive | 7 | Pulmonary edema |
| J84 | negative | 0 | Pulmonary fibrosis |
| J84 | positive | 3 | Pulmonary fibrosis |
| J90 | negative | 1 | pleural effusion |
| J90 | positive | -12 | pleural effusion |
| J96 | negative | -1 | Respiratory failure |
| J96 | positive | 5 | Respiratory failure |
| J98 | negative | -2 | Respiratory disorder, unspecified |
| J98 | positive | 7 | Respiratory disorder, unspecified |
| K21 | negative | 1 | Gastro-esophageal reflux disease with esophagitis |
| K21 | positive | -2 | Gastro-esophageal reflux disease with esophagitis |
| K31 | negative | 1 | Other diseases of stomach and duodenum |
| K31 | positive | -12 | Other diseases of stomach and duodenum |
| K42 | negative | 1 | Umbilical hernia with obstruction |
| K42 | positive | -31 | Umbilical hernia with obstruction |
| K44 | negative | -1 | Diaphragmatic hernia with obstruction, without gangrene |
| K44 | positive | 10 | Diaphragmatic hernia with obstruction, without gangrene |
| K52 | negative | 1 | Other noninfective gastroenteritis and colitis |
| K52 | positive | -9 | Other noninfective gastroenteritis and colitis |
| K59 | negative | 1 | Other functional intestinal disorders |
| K59 | positive | -5 | Other functional intestinal disorders |
| K63 | negative | 0 | Other diseases of intestine |
| K63 | positive | 1 | Other diseases of intestine |
| K74 | negative | -1 | Fibrosis and cirrhosis of liver |
| K74 | positive | 16 | Fibrosis and cirrhosis of liver |
| K76 | negative | 0 | Other diseases of pancreas |
| K76 | positive | -3 | Other diseases of pancreas |
| K80 | negative | 0 | Cholelithiasis |
| K80 | positive | 3 | Cholelithiasis |
| K86 | negative | 3 | Other diseases of pancreas |
| K86 | positive | -57 | Other diseases of pancreas |
| K92 | negative | 0 | Gastrointestinal hemorrhage |
| K92 | positive | 4 | Gastrointestinal hemorrhage |
| L02 | negative | 0 | Cutaneous abscess, furuncle and carbuncle |
| L02 | positive | -4 | Cutaneous abscess, furuncle and carbuncle |
| L03 | negative | 0 | Cellulitis |
| L03 | positive | -2 | Cellulitis |
| L98 | negative | 0 | pressure ulcers, also known as bed sores, pressure sores or decubitus ulcers |
| L98 | positive | 2 | pressure ulcers, also known as bed sores, pressure sores or decubitus ulcers |
| M | negative | 1 |  |
| M | positive | -1 |  |
| M12 | negative | 0 | arthropathy |
| M12 | positive | 3 | arthropathy |
| M19 | negative | 0 | Osteoarthritis |
| M19 | positive | 1 | Osteoarthritis |
| M43 | negative | 0 | spondylolisthesis |
| M43 | positive | -1 | spondylolisthesis |
| M48 | negative | 1 | Spinal stenosis |
| M48 | positive | -14 | Spinal stenosis |
| M51 | negative | 0 | Intervertebral disc disorder |
| M51 | positive | -1 | Intervertebral disc disorder |
| M62 | negative | 0 | Other disorders of muscle |
| M62 | positive | 1 | Other disorders of muscle |
| M85 | negative | 0 | Other disorders of bone density and structure |
| M85 | positive | -4 | Other disorders of bone density and structure |
| M89 | negative | 1 | Disorder of bone |
| M89 | positive | -19 | Disorder of bone |
| N13 | negative | 0 | Obstructive and reflux uropathy |
| N13 | positive | -3 | Obstructive and reflux uropathy |
| N17 | negative | -4 | Acute kidney failure |
| N17 | positive | 14 | Acute kidney failure |
| N18 | negative | 6 | Chronic kidney disease |
| N18 | positive | -34 | Chronic kidney disease |
| N20 | negative | 2 | Calculus of kidney and ureter |
| N20 | positive | -34 | Calculus of kidney and ureter |
| N28 | negative | 2 | Other disorders of kidney and ureter, not elsewhere classified |
| N28 | positive | -13 | Other disorders of kidney and ureter, not elsewhere classified |
| N32 | negative | 1 | Other disorders of bladder |
| N32 | positive | -17 | Other disorders of bladder |
| N39 | negative | 1 | Urinary tract infection |
| N39 | positive | -4 | Urinary tract infection |
| N40 | negative | 0 | Benign Prostatic Hyperplasia with Lower Urinary Tract Symptoms |
| N40 | positive | 2 | Benign Prostatic Hyperplasia with Lower Urinary Tract Symptoms |
| No_alcohol | negative | 11 |  |
| No_alcohol | positive | -11 |  |
| No_Substance_use | negative | 14 |  |
| No_Substance_use | positive | -5 |  |
| Non-Hispanic/Latino | negative | 35 |  |
| Non-Hispanic/Latino | positive | -5 |  |
| O09 | negative | 3 | Supervision of high risk pregnancy |
| O09 | positive | -41 | Supervision of high risk pregnancy |
| O26 | negative | 0 | Conditions predominantly related to pregnancy |
| O26 | positive | 6 | Conditions predominantly related to pregnancy |
| O99 | negative | 1 | Other maternal diseases classifiable elsewhere but complicating pregnancy, childbirth and the puerperium |
| O99 | positive | -7 | Other maternal diseases classifiable elsewhere but complicating pregnancy, childbirth and the puerperium |
| R00 | negative | 1 | Abnormalities of heart beat |
| R00 | positive | -4 | Abnormalities of heart beat |
| R05 | negative | -45 | Cough |
| R05 | positive | 36 | Cough |
| R06 | negative | -1 | Dyspnea |
| R06 | positive | 1 | Dyspnea |
| R07 | negative | -1 | Pain in throat and chest |
| R07 | positive | 1 | Pain in throat and chest |
| R10 | negative | 2 | Abdominal and pelvic pain |
| R10 | positive | -3 | Abdominal and pelvic pain |
| R11 | negative | 1 | Nausea and vomiting |
| R11 | positive | -4 | Nausea and vomiting |
| R13 | negative | 0 | Dysphagia |
| R13 | positive | 2 | Dysphagia |
| R14 | negative | 0 | Abdominal distension (gaseous) |
| R14 | positive | -2 | Abdominal distension (gaseous) |
| R16 | negative | 1 | Hepatomegaly and splenomegaly |
| R16 | positive | -9 | Hepatomegaly and splenomegaly |
| R18 | negative | 1 | Ascites |
| R18 | positive | -19 | Ascites |
| R19 | negative | 1 | Symptoms, signs and abnormal clinical and laboratory findings, not elsewhere classified |
| R19 | positive | -3 | Symptoms, signs and abnormal clinical and laboratory findings, not elsewhere classified |
| R20 | negative | -1 | Disturbances of skin sensation |
| R20 | positive | 8 | Disturbances of skin sensation |
| R21 | negative | 1 | Rash and other nonspecific skin eruption |
| R21 | positive | -7 | Rash and other nonspecific skin eruption |
| R22 | negative | 0 | Localized swelling, mass and lump of skin and subcutaneous tissue |
| R22 | positive | 2 | Localized swelling, mass and lump of skin and subcutaneous tissue |
| R29 | negative | 0 | Other symptoms and signs involving the nervous and musculoskeletal systems |
| R29 | positive | 1 | Other symptoms and signs involving the nervous and musculoskeletal systems |
| R30 | negative | 0 | Dysuria |
| R30 | positive | -2 | Dysuria |
| R31 | negative | 0 | Hematuria |
| R31 | positive | 1 | Hematuria |
| R32 | negative | 0 | Unspecified urinary incontinence |
| R32 | positive | -1 | Unspecified urinary incontinence |
| R33 | negative | -1 | Retention of urine |
| R33 | positive | 14 | Retention of urine |
| R35 | negative | -1 | polyurea |
| R35 | positive | 7 | polyurea |
| R39 | negative | 0 | Other difficulties with micturition |
| R39 | positive | 1 | Other difficulties with micturition |
| R41 | negative | -1 | Other symptoms and signs involving cognitive functions and awareness |
| R41 | positive | 6 | Other symptoms and signs involving cognitive functions and awareness |
| R42 | negative | 0 | Dizziness and Giddiness |
| R42 | positive | -2 | Dizziness and Giddiness |
| R45 | negative | 2 | Symptoms and signs involving emotional state |
| R45 | positive | -21 | Symptoms and signs involving emotional state |
| R47 | negative | 1 | Unspecified speech disturbances |
| R47 | positive | -28 | Unspecified speech disturbances |
| R50 | negative | -5 | Fever |
| R50 | positive | 22 | Fever |
| R52 | negative | -1 | Pain |
| R52 | positive | 7 | Pain |
| R53 | negative | 0 | Malaise and fatigue |
| R53 | positive | 1 | Malaise and fatigue |
| R55 | negative | 0 | Syncope and collapse |
| R55 | positive | -1 | Syncope and collapse |
| R56 | negative | 0 | Convulsions |
| R56 | positive | -1 | Convulsions |
| R59 | negative | 2 | Enlarged lymph nodes |
| R59 | positive | -15 | Enlarged lymph nodes |
| R65 | negative | 0 | Systemic inflammation and infection |
| R65 | positive | 2 | Systemic inflammation and infection |
| R73 | negative | 0 | Elevated blood glucose level |
| R73 | positive | 2 | Elevated blood glucose level |
| R78 | negative | 0 | Findings of drugs and other substances, not normally found in blood |
| R78 | positive | 1 | Findings of drugs and other substances, not normally found in blood |
| R79 | negative | 3 | Other abnormal findings of blood chemistry |
| R79 | positive | -19 | Other abnormal findings of blood chemistry |
| R80 | negative | 0 | Persistent proteinuria |
| R80 | positive | 1 | Persistent proteinuria |
| R82 | negative | 0 | Other and unspecified abnormal findings in urine |
| R82 | positive | 1 | Other and unspecified abnormal findings in urine |
| R92 | negative | 0 | Abnormal and inconclusive findings on diagnostic imaging of breast |
| R92 | positive | 4 | Abnormal and inconclusive findings on diagnostic imaging of breast |
| R93 | negative | 2 | Abnormal findings on diagnostic imaging of other specified body structures |
| R93 | positive | -8 | Abnormal findings on diagnostic imaging of other specified body structures |
| R94 | negative | 0 | Abnormal results of liver function studies |
| R94 | positive | 2 | Abnormal results of liver function studies |
| S00 | negative | 1 | Superficial injury of head |
| S00 | positive | -19 | Superficial injury of head |
| S09 | negative | 1 | Other and unspecified injuries of head |
| S09 | positive | -27 | Other and unspecified injuries of head |
| T14 | negative | 1 | injury |
| T14 | positive | -13 | injury |
| T81 | negative | 0 | Complications of procedures, not elsewhere classified |
| T81 | positive | -2 | Complications of procedures, not elsewhere classified |
| T82 | negative | -1 | Complications of cardiac and vascular prosthetic devices, implants and grafts |
| T82 | positive | 11 | Complications of cardiac and vascular prosthetic devices, implants and grafts |
| White | negative | 21 |  |
| White | positive | -26 |  |
| Z33 | negative | 0 | Pregnancy |
| Z33 | positive | -6 | Pregnancy |
| Z37 | negative | -1 | Outcome of delivery |
| Z37 | positive | 27 | Outcome of delivery |
| Z3A | negative | 6 | Pregnancy |
| Z3A | positive | -61 | Pregnancy |
| Z74 | negative | -1 | Problems related to care provider dependency |
| Z74 | positive | 3 | Problems related to care provider dependency |
| Z78 | negative | 1 | Other long term (current) drug therapy |
| Z78 | positive | -12 | Other long term (current) drug therapy |
| Z90 | negative | 2 | Acquired absence of organs, not elsewhere classified |
| Z90 | positive | -12 | Acquired absence of organs, not elsewhere classified |
| Z95 | negative | 1 | Presence of cardiac and vascular implants and grafts |
| Z95 | positive | -17 | Presence of cardiac and vascular implants and grafts |
| Z98 | negative | 0 | Other postprocedural states |
| Z98 | positive | 2 | Other postprocedural states |
| Z99 | negative | 1 | Dependence on enabling machines and devices |
| Z99 | positive | -11 | Dependence on enabling machines and devices |

**REFERENCES**

Yurdakul, B., and Naranjo, J. (2019). Statistical Properties of the Population Stability Index. *Journal of Risk Model Validation* 14(4).
